# 

**Published:** 1904-03-26

**Authors:** 


### CONTENTS.

**The Homeless Poor** - 457
**Ambidexterity** - 458
**Annotations** - 459
**Notes and News** - 460
**Hospital Clinics and Medical Progress—**
The Physiology of Simultaneous Ambidextral Work - 461
Black Urine - 462
Post-Basal Meningitis due to Pneumococcus Lanceolatus - 463
Extraction of Needles after Location with the X-Rays - 463
The Cause of Rickets - 464
Progress in Pediatrics - 464
Progress in Cerebral, Spinal, and Nerve Surgery - 465
Progress in Diseases of the Nervous System - 466
**Hospital Administration—**
Hospital Meetings - 468
Practical Departments - 471
**The Student of Medicine** - 472

*NURSING SECTION.*

**Notes on News from the Nursing World—** Queen Alexandra's Imperial Military Nursing Service—The Qualifications for American Army Nurses—The New Matron of the Royal Prince Alfred Hospital—A Nurses' Home for University College Hospital—Gold Medal for a Matron—An Erroneous Assumption—A Home for the City of London Chest Hospital—A Gloomy Prospect at Worcester—Up-Country Nursing in India—Nurses as Private Detectives—A Touching Incident at Shoreditch—An Admirable Suggestion at Shrewsbury—The Matronships of Darenth and Belmont Asylums—Starting a Nurse in the Right Way—The Duty of Torquay—Short Items - 345-348
**The Nursing Outlook** - 349
**Lectures upon the Nursing of Mental Diseases** 350
**State Registration of Trained Nurses** - 351
**Choosing the New Matron of the Royal Prince Alfred Hospital, Sydney** - 353
**The Irish Nurses' Association** - 353
**Everybody's Opinion** - 354
**Appointments** - 354
**Novelties for Nurses** - 354
**Echoes from the Outside World** - 355
**Notes and Queries** - 3[illegible]6
**For Reading to the Sick** - 356

